# Longitudinal spin separation of light and its performance in three-dimensionally controllable spin-dependent focal shift

**DOI:** 10.1038/srep20774

**Published:** 2016-02-17

**Authors:** Sheng Liu, Peng Li, Yi Zhang, Xuetao Gan, Meirong Wang, Jianlin Zhao

**Affiliations:** 1 Key Laboratory of Space Applied Physics and Chemistry, Ministry of Education and Shaanxi Key Laboratory of Optical Information Technology, School of Science, Northwestern Polytechnical University, Xi’an 710129, China

## Abstract

Spin Hall effect of light, which is normally explored as a transverse spin-dependent separation of a light beam, has attracted enormous research interests. However, it seems there is no indication for the existence of the longitudinal spin separation of light. In this paper, we propose and experimentally realize the spin separation along the propagation direction by modulating the Pancharatnam-Berry (PB) phase. Due to the spin-dependent divergence and convergence determined by the PB phase, a focused Gaussian beam could split into two opposite spin states, and focuses at different distances, representing the longitudinal spin separation. By combining this longitudinal spin separation with the transverse one, we experimentally achieve the controllable spin-dependent focal shift in three dimensional space. This work provides new insight on steering the spin photons, and is expected to explore novel applications of optical trapping, manipulating, and micromachining with higher degree of freedom.

Spin Hall effect of light (SHEL), a typical manifestation of photonic spin-orbit interaction, has aroused researchers’ enormous interest after its theoretical prediction^1^ and first experimental observation^2^. As an analogy to spin Hall effect of electronic system, SHEL has potentials to reveal novel optical applications. It has been demonstrated that a light beam presents transverse separation of the opposite photonic spin states (circular polarizations) during its propagation, when the spin and orbital angular momenta (SAM and OAM) are mutually coupled. To trigger SHEL, a variety of methods and devices have been proposed for inducing spin-orbit coupling of a light beam, such as, refracting and reflecting at the interfaces of media^2^,^3^,^4^ or metamaterial^5^,^6^,^7^, propagating along a coiled trajectory in glass cylinder^8^, tilted reference frame with respect to the propagation direction^9^,^10^, symmetry-breaking vector beam^11^,^12^,^13^, and photonic graphene^14^, etc. Traditionally, the reported SHEL can be divided into two categories according its physical mechanism: one arising from the so called Imbert-Fedorov (IF) effect^15^,^16^,^17^, which describes the spin-dependent shift of light reflected or refracted at the media surface; the other one from the purely geometric nature of tilted reference frame, named geometric spin Hall effect of light^9^,^10^. Recently, it was reported that the Pancharatnam-Berry (PB) phase can also be employed to realize the enhanced spin separation of light^18^,^19^, which could be observed directly without using weak measurement technology^2^.

However, all the reported SHEL merely demonstrated the transverse separation of spin states as far as we know. And it seems there is no indication for the existence of the longitudinal SHEL, i.e. the spin separation along the propagation direction of light. In this paper, we experimentally realize the longitudinal spin separation of light by modulating the PB phase. After passing through a polarization-transporting system, a focused Gaussian beam splits into two beams with opposite spin states and focuses at different distances, representing the longitudinal spin-dependent focal shift. Combining this longitudinal spin separation with the transverse one, we experimentally achieve the controllable spin-dependent focal shift in three dimensional space.

## Results

### Theoretical analysis

Let us first briefly analyze the geometric phase produced by the variation of polarizations, i.e. PB phase. It was point out that when a light beam undergoes a polarization transformation along a close cycle on the Poincaré sphere, it acquires an additional geometric phase, named PB phase^20^,^21^,^22^. The PB phase is related to the solid angle subtended by the corresponding geodesic triangle on the Poincaré sphere.

**Figure 1:**
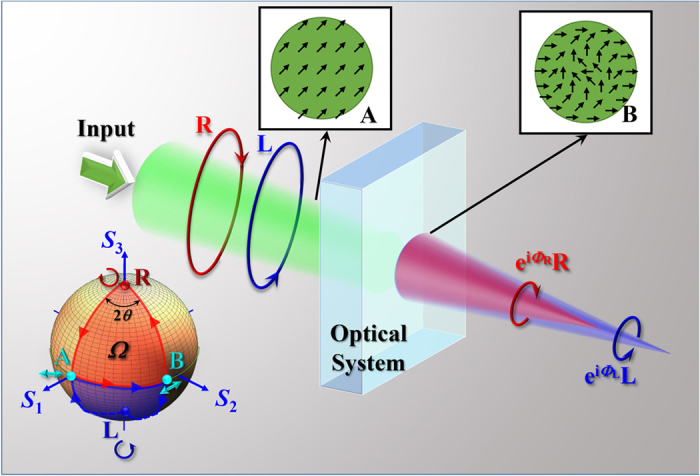
Schematic of spin separation and Pancharatnam-Berry (PB) arising from polarization transformation. A focused Gaussian beam with linear polarization **A** passes through an inhomogeneous optical system, changes its polarization state to **B**, and then splits into a pair of spin states attached with additional different PB phase *Φ*_*L*_ and *Φ*_*R*_. By properly selecting the PB phases, the two spin states would focus at different location. Insert: Schematic illustration of polarization transformation in the Poincaré spheres.

A simple example should make the PB phase easier to understand. For a light beam with polarization state **A**, assuming it is linearly polarized along *x* axis, its polarization state **e**_**A**_ (the unit vector of polarization state **A**) is described by **e**_**A**_ = **e**_*x*_, where **e**_*x*_ denotes the unit vector along the *x* axis. Generally, this light beam can be considered as a composition of two spin states **L** and **R**, corresponding to the left-handed (LH) and right-handed (RH) circular polarizations, respectively. Namely, 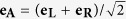, where **e**_**L**_ and **e**_**R**_ respectively denote the unit vectors of LH and RH polarizations, meeting 
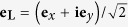
 and 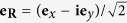, where **e**_*y*_ denotes the unit vector along the *y* axis. After passing through an optical system (e.g. wave plate, or polaroid), as shown in Fig. 1, the polarization state of the light beam is changed into **B**. If **B** is another linear polarization state and its polarization direction along angle *θ*, it meets **e**_**B**_ = cos *θ***e**_*x*_ + sin *θ***e**_*y*_, where **e**_**B**_ is the unit vector of polarization state **B**. This light beam could be also decomposed in two spin states *L* and *R*, i.e. 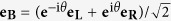. Obviously, after polarization transformation from **A** to **B**, the LH and RH components are appended phase −*θ* and *θ*, respectively, which can be considered as the PB phase.

In the whole process of the polarization transformation, for the RH polarization state, it can be considered to experience a cyclic polarization change (see the red arrows in the left-bottom of Fig. 1): from **R** to **A** by directly projecting, then transporting to **B**, and finally projecting back to **R**. The PB phase is equal to minus half the solid angle Ω subtended by the geodesic triangle **R** → **A** → **B** → **R**. The sign of Ω depends on the order of polarization transformation^20^. In this case, the PB phase is equal to *θ*. Similarly for the LH polarization state, it experiences the polarization changes as **L** → **A** → **B** → **L** (see the blue arrows in the left-bottom of Fig. 1), and obtains a PB phase −*θ*.

In a general case, for arbitrary polarization states **A** and **B**, the PB phase can also be generated by the polarization transformation. Supposing the polarization transformation between **A** and **B** is achieved through an inhomogeneous wave plate or subwavelength grating^19^,^23^, **B** becomes a space-variant polarization state, e.g. **e**_**B**_**(x**, **y)** = cos *θ*(*x, y*)**e**_*x*_ + sin *θ*(*x, y*)**e**_*y*_. Accordingly, the PB phase gets a space-variant distribution, which can modulate the wave front and tune the propagation of light. It is therefore convenient to control the propagations of the two spin states by designing the space-variant PB phases.

To consist with our experimental condition, a light beam linearly polarized along the angle of *θ*_0_ (i.e. **e**_*A*_ = cos *θ*_0_**e**_*x*_ + sin *θ*_0_**e**_*y*_), which represents the most common laser beam, is selected as the input beam. And then the input vector field can be described by **E**_**A**_ = *E*_0_**e**_*A*_, where *E*_0_ is the complex amplitude distribution. The LH and RH components of this linear polarized beam have the initial phase −*θ*_0_ and *θ*_0_, respectively. Namely. 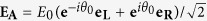. We denote the PB phases acquired by LH and RH states as *Φ*_*L*_(*x, y*) and *Φ*_*R*_(*x, y*), respectively. For simplicity, we neglect the loss during polarization transformation, and the light beam after varying its polarization state could be expressed as


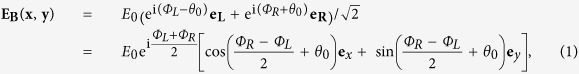


Equation (1) indicates that the optical system converts the input beam **E**_**A**_ to the linearly polarized **E**_**B**_ with the polarization direction along an angle (*Φ*_*R*_ − *Φ*_*L*_)/2 + *θ*_0_, appending an additional phase retardation (*Φ*_*R*_ + *Φ*_*L*_)/2. Meanwhile, the intensity profile of the light beam is not changed during this conversion.

There are many methods to change polarization state from **A** to **B**, such as via inhomogeneous wave plate or subwavelength grating^19^,^23^, and even optical systems^18^,^24^,^25^. It has to be emphasized that the phase term (*Φ*_*R*_ + *Φ*_*L*_)/2 is mainly originated from the phase retardation of optical elements during the polarization variation. In general optical systems, this phase term is a constant and hard to control. For convenient, we set that (*Φ*_*R*_ + *Φ*_*L*_)/2 = 0, and make *Φ*_*L*_ = −*Φ*_*R*_ = *Φ*(*x, y*) + *θ*_0_. Then the polarization transformation process could be simplified as


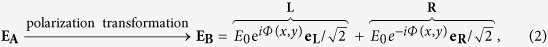


where **E**_**B**_ = *E*_0_(cos *Φ***e**_*x*_ − sin *Φ***e**_*y*_) denotes a space-variant linear polarization with its local polarization direction along the azimuth angle −*Φ*. Equation (2) indicates that the input beam **E**_**A**_ is converted to **E**_**B**_, and decomposed into two spin components with different PB phases *Φ*(*x, y*) and −*Φ*(*x, y*). To separate these two spin components, *Φ*(*x, y*) need to be selected as a special distribution.

### Spin-dependent divergence and convergence

One of the most typical selection of PB phase is the phase factor of a tilted plane wave, e.g. *Φ* = *k*_*x *_*x*, as reported in ref. 19. With the modulation of the PB phase, the two spin components obtain two mutually conjugate tilted phases (i.e. 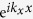 and 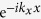), and would separate transversely with each other during propagating. This type of transverse separation is considered as a giant SHEL which can be conveniently controlled by the PB phase.

Another typical selection of *Φ* is the phase factor of a spherical wave, i.e. *Φ* = *αr*^2^, where *r* denotes the radial coordinate, and *α* is a nonzero constant. In this case, the two output spin states would carry phase factors of converging and diverging spherical waves 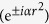, respectively. If *α* > 0, the LH and RH polarization states are divergent and convergent, respectively. Whereas if *α* < 0 they go the opposite.

**Figure 2:**
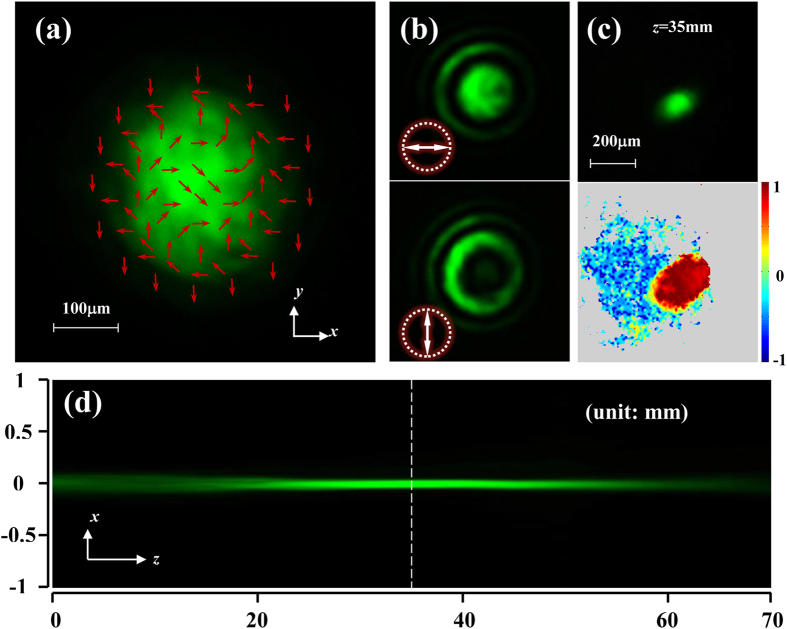
Spin-dependent divergence and convergence. (**a**) Input beam with polarization direction marked with red arrowheads. (**b**) Horizontally and vertically polarized components of **E**_**B**_. (**c**) Output beam (top), and the corresponding distribution of Stokes parameter *s*_3_ (bottom). (**d**) Side view of experimentally recorded beam propagation.

To experimentally realize the spatially inhomogeneous polarization transformation, a Sagnac interferometer^24^,^25^ is employed. A linearly polarized Gaussian beam (from Ar+ laser, with wavelength of *λ* = 514.5 nm) with a waist diameter about 300 μm is input to the interferometer, with its intensity distribution shown in Fig. 2(a). To ensure the interferometer only changes the polarization of the input beam without the variation of intensity profile, a pair of imaging lens are set at the input and output places with the conjugate imaging distances. In the interferometer, a spatial light modulator (SLM) is used to control the PB phase during the polarization transformation. Here, we design a PB phase with *α* = 1.65 × 10^8^ m^−2^, and the polarization directions of the output beam (**E**_**B**_) are depicted as the red arrowheads in Fig. 2(a), representing clear radial-variant polarization. Considering **E**_**B**_ is space-variant linearly polarized, we use an analyzer to determine the polarization distribution, as shown in Fig. 2(b), where the top and bottom display to some extent the intensity distribution of 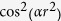 and 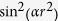 for horizontally (**e**_*x*_) and vertically (**e**_*y*_) polarized components, respectively.

Subsequently, the LH and RH components would obtain diverging and converging phase factors, representing defocusing and focusing propagations, respectively. To monitor the propagating process of light, the output field is recorded by a CCD camera, which is moved to different locations step by step. Figure 2(d) shows the experimental result of the side view of beam propagation within 70 mm in the *x-z* plane. After polarization transformation, the Gaussian beam experiences focusing with the minimum beam spot at *z* = 35 mm. To confirm the polarization of the output beam, the Stokes parameter *s*_3_ is measured by introducing a *λ*/4 plate and an analyzer^26^. The measured result at the propagation distance *z* = 35 mm is shown in the bottom of Fig. 2(c), where the blue (*s*_3_ = −1) and red (*s*_3_ = 1) regions represent LH and RH polarizations, respectively. It is clearly seen that the focusing spot is RH polarized, in accordance with the expectation.

This phenomenon represents another type of spin-dependent separation similar as our previous work^25^: one spin state is weakened while the other one is enhanced and manifests itself. It is important to note that the total integrated intensity for each spin state is not changed during propagation, and the total spin angular momentum is still conserved in any propagation plane. However, the spin-dependent divergence and convergence lead to the huge unbalance of the energy density between the two spin states. Namely, there still exists weak (divergent) LH polarized field superimposed with the focused RH component. As a result, the Stokes parameter *s*_3_ in Fig. 2(c) is not exactly equal to 1 for the focused beam. However, the divergent LH state is too weak (several orders of magnitude weaker than that of RH state) to influence the focused RH state. Actually, higher value of |*α*| could result in more convergent RH component and more divergent LH component. And then, the value of *s*_3_ of output beam is much closer to 1, namely, a more pure RH state would be formed. Likewise, if it is expected to focus the LH spin state, the constant *α* should be set as a negative value. And more importantly, the focusing distance *f* can be changed with the value of |*α*| according to


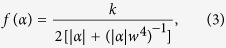


where *k* is the wave vector, and *w* is the initial beam radius.

### Longitudinal spin separation

As a matter of fact, the radial-variant polarization in Fig. 2 can play a greater role in the longitudinal separation of spin states. Here, we set *E*_0_ as a focused Gaussian profile [with the phase factor 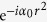, *α*_0_ > 0]. After the similar PB phase modulation as demonstrated in Fig. 2, the focused Gaussian beam would split into a pair of spin states associated with additional divergent and convergent phase factor 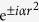, respectively. According to the analysis in the above section, the convergent phase factor will speed up the focusing process of the Gaussian beam, while the divergent one will make it slow down. Therefore, the two spin states [with the phase factors 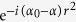 and 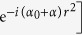 will separately focus at different distances. The performance of this spin-dependent focal shift would resemble a novel “longitudinal SHEL”, namely, the longitudinal spin separation.

**Figure 3:**
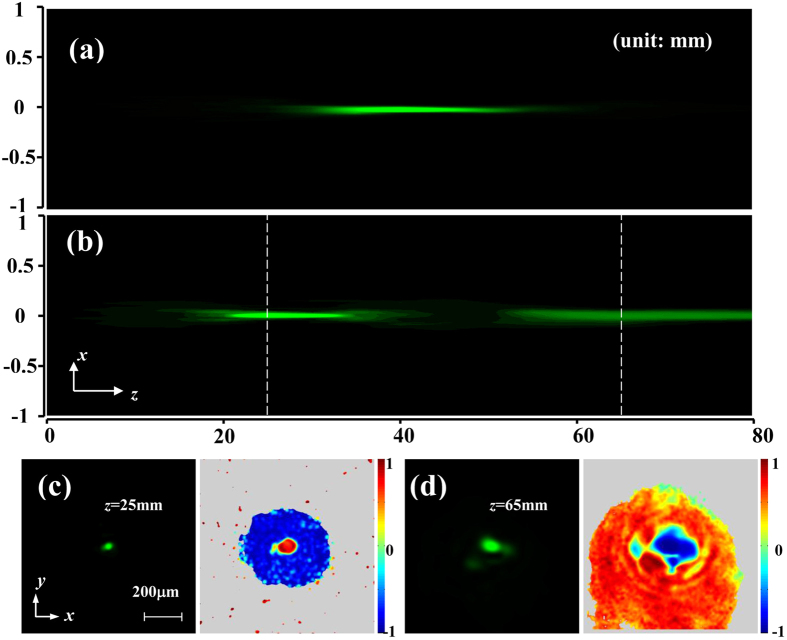
Longitudinal separation of spin states. (**a**,**b**) Side view of the propagations of a focused linearly polarized Gaussian beam before and after polarization transformation. (**c**,**d**) Output beam (left) and the corresponding *s*_3_ distribution (right) at *z* = 25 mm and 65 mm.

Figure 3 depicts the experimental verification of the longitudinal spin separation. The input Gaussian beam has a diameter about 360 μm, and the convergent phase factor is realized by a lens with focal length of 38 mm (the calculated *α*_0_ is 1.55 × 10^8^ m^−2^), as shown in Fig. 3(a). In the Sagnac interferometer, we design a phase factor of spherical wave with *α* = 0.75 × 10^8^ m^−2^ on the SLM. Subsequently, the LH and RH components of the beam after the Sagnac interferometer would attach the phase factors of spherical wave 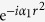 and 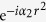, respectively, where *α*_1_ = 0.8 × 10^8^ m^−2^, and *α*_2_ = 2.3 × 10^8^ m^−2^. According to Eq.(3), these two spin components would be focused at *z* = 66.4 mm and 26.1 mm, respectively. The measurement result of the propagation process of the output beam is depicted in Fig. 3(b). It is evident that the Gaussian beam splits longitudinally into two focusing spots at different distances (about 25 mm and 65 mm, respectively) which are nicely in accord with the calculations. To confirm the polarization states of the double-focusing beam, the Stokes parameters *s*_3_ at *z* = 25 mm and 65 mm are measured, as displayed in the right of Fig. 3(c,d), respectively. It is shown that the two focusing spots at *z* = 25 mm and 65 mm are RH and LH polarized, respectively, which indicates the realization of a longitudinal spin separation.

**Figure 4:**
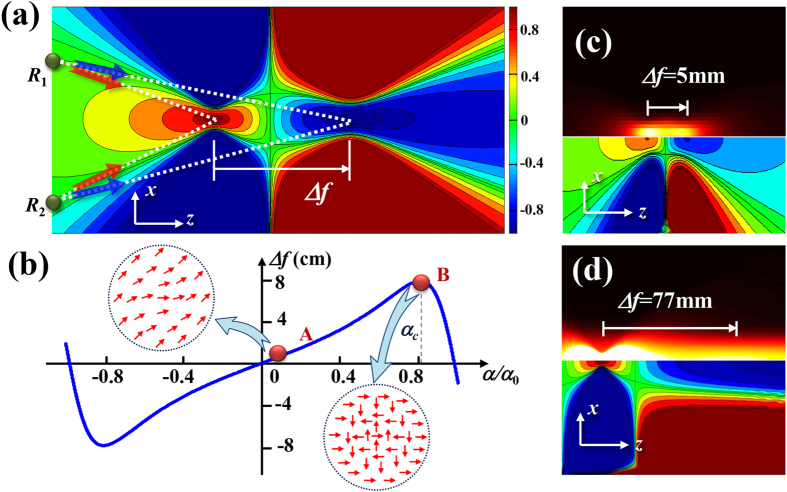
Properties of longitudinal separation of spin states. (**a**) Calculated *s*_3_ at *x*–*z* plane. (**b**) Curve of Δ*f* vs. *α*/*α*_0_ for the experimental parameters. (**c**,**d**) Intensity (top) and *s*_3_ distributions (bottom) at *x*–*z* plane of points A and B in (**b**), respectively. Inserts in (**b**): transformed polarization distributions corresponding to points A and B.

Note that, the separation distance is mainly determined by the parameter *α*. However, to realize the longitudinal spin separation, the divergent phase factor arising from the PB phase can not offset the convergence of the input focused Gaussian beam. Namely, it must satisfy *α* < *α*_0_. Otherwise, the second focusing in Fig. 3(b) will be never formed, and the corresponding spin state will be drowned out by the light field. For a fixed focused Gaussian beam, the controllability of the spin separation can be realized by changing the parameter *α* of the PB phase. Here, we define the spin separation Δ*f* = *f*(*α*_0_ − *α*) − *f*(*α*_0_ + *α*) as the distance between the focusing spots (at the locations of the maximum intensities) of LH and RH components [as shown in Fig. 4(a)], where the negative value means that the RH polarized focusing spot is farther than the LH one. Figure 4(b) depicts the curve of spin separation versus *α*. It can be seen that when |*α*| is smaller than a critical value *α*_*c*_ (determined by the beam size and the initial convergent phase factor), Δ*f* increases with *α* monotonously. Once *α* exceeds this critical value, the waist position of the Gaussian beam with phase factor 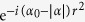 would rapidly move back to *z* = 0 plane as *α* increasing. As a result, the separation is sharply reduced. When *α* is a small value [e.g. point A in Fig. 4(b)], the separation is too small to be distinguished as shown in Fig. 4(c), and make it difficult to determine the locations of the two focal points. However, it might provide a novel technology to extend the focal depth of an image system^27^. While if *α* is set as a large value [e.g. point B in Fig. 4(b)], the second focusing spot is too wide and weak [see Fig. 4(d)] to manifest itself. Therefore, to realized a visible longitudinal spin separation, the parameter *α* need to be selected in an appropriate range (generally, *α*/*α*_0_ is around 0.5).

The polarization of the modulated Gaussian beam also presents a propagation-varied polarization, as shown in Fig. 4(a). For example, the polarization is changed from RH polarization (at the first focal plane) to linear one (at the distance that the two components have the same intensity profile), and then to LH one (at the second focal plane), similar to the polarization distribution of transverse SHEL. Due to the focusing effect, the two spin states can manifest themselves at different distances.

### Spin-dependent focal shift in three dimensional space

Considering that the PB phase can be selected as any expected phase, we can conveniently control the propagation of output beam. The transverse and longitudinal separations of spin states can be achieved simultaneously by employing the portfolio of the phase factors of plane and spherical waves. At this point, we can realize the separation of spin states with three degrees of freedom (along *x, y* and *z* axes, respectively). For a focused Gaussian beam, if setting the PB phase as


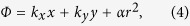


we can control the spin-dependent separation along *x, y*, and *z* axes arbitrarily by appropriately setting the three parameters *k*_*x*_, *k*_*y*_, and *α*, respectively. And the sign of the above three parameters could reverse the shift directions of the LH and RH components. It can be concluded that the LH and RH components are focused at the coordinates (*x*_*L*_, *y*_*L*_, *z*_*L*_) and (*x*_*R*_, *y*_*R*_, *z*_*R*_), respectively, where 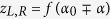, *x*_*L*,*R*_ = *k*_*x*_*z*_*L*,*R*_/*k*, and *y*_*L*,*R*_ = *k*_*y*_*z*_*L*,*R*_/*k*.

**Figure 5:**
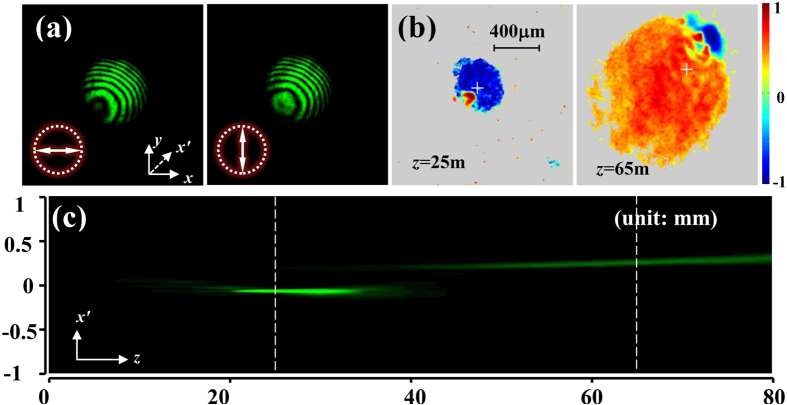
Spin-dependent separation in three dimensional space. (**a**) Horizontally and vertically polarized components of *E*_*B*_. (**b**) Distributions of *s*_3_ at *z* = 25 mm and 65 mm. (**c**) Side view of the propagation in *x*′–*z* plane.

To experimentally verify the three dimensional spin separation, we set *k*_*x*_ = *k*_*y*_ = 5.0 × 10^4^ m^−1^, and *α* = 0.75 × 10^8^ m^−2^, and the input beam is chosen as the focused Gaussian beam used in Fig. 3. After polarization transformation, the horizontally and vertically polarized components of *E*_*B*_ are shown in Fig. 5(a). By observing the propagation process, we can find the beam is focused at *z* = 25 mm and 65 mm. According to the corresponding *s*_3_ depicted in Fig. 5(b), we obtain that the two focusing spots are RH and LH polarized, respectively. It also can be obviously seen the two spin states leave the initial beam centre [marked by white crosses as shown in Fig. 5(b)] along opposite directions, performing the transvers shift both along *x* and *y* axes. To depict the propagation process, an axis, denoted as *x*′ axis, is built with the orientation from the coordinate (*x*_1_, *y*_1_) of the first focusing spot to the coordinate (*x*_2_, *y*_2_) of the second one. It is measured that *x*_1_ = *y*_1_ = −0.1 mm and *x*_2_ = *y*_2_ = 0.25 mm. The propagation process in view of *x*′–*z* plane is shown in Fig. 5(c)], which evidently shows that two focusing spots respectively appear at *z* = 25 mm and 65 mm, with opposite transverse shifts along *x*′ axis.

## Discussion

The above assumption that the input beam is a focused one is a much common setting in the actual situation: passing through a lens. And it has to be emphasized that the longitudinal spin separation of light occurs whether the polarization transformation happens after or before this lens, because the spin-dependent divergence and convergence can work in the both cases. Generally, we can firstly transform the polarization of a beam and then focus it via a lens. For the PB phase described by Eq. 4, it provides the general idea to simultaneously focus two spin states to spots at customizable longitudinal and transverse locations. Actually, the similar polarization-dependent focal shift has been reported with different methods^27^,^28^, and the potential applications are also valid for the scheme proposed in this paper. For example for the optical trapping of three-dimensional structures^29^, we can focus one vortex beam with an object lens into two different spatial locations in three-dimensional space to drive and rotate different micro-objects.

It needs to be emphasized that the longitudinal spin separation is actually an overall effect of the inhomogeneous transverse SHEL. As depicted in Fig. 4(a), the secondary wave of an arbitrary point at the output plane *z* = 0 (e.g. point R_1_, or R_2_) would transversely split into a pair of spin states, where the photons with LH and RH polarizations travel in the directions of the first and second focal points, respectively. As a result, the photon energy of LH and RH components are gathered in the two focal points, respectively, and exhibit the longitudinal spin separation. Importantly, the inhomogeneous transverse spin separation is induced by the nonuniform gradient of the PB phase, i.e. the phase gradient is not a constant. It is naturally to deduce that a PB phase with nonuniform gradient, rather than the phase factor of a spherical wave, can also lead to the some level of convergence of spin states at different spatial locations, and represents the spin separation in three dimensional space. This inhomogeneous spin-separation effect is very similar to the polarization-dependent shift of a light beam of finite transverse extent^30^. It is considered that the spatial or angular shift for a reflecting Gaussian beam is the weighted sum of the subtle shifts of different plane wave components, which are also inhomogeneous, and give rise to the deflection of the reflection. Nevertheless, in our scheme, we provide more flexibility and controllability for the inhomogeneous spin separation (angular shift) by employing the PB phase.

The second thing to be emphasized is that the separation scales of the spin states demonstrated in this paper is far greater than that of the traditional SHEL, and can be considered as a macro effect of light. During the spin-dependent focal shift in longitudinal direction or in three-dimensional space, the variation of the spin direction of light might give rise to SHEL, however, of which the magnitude was too tiny to stand out from the macro effect. Thus for the spin separation along the longitudinal axis, the measurement is done at the millimeter scale accuracy, which is comparable to the focal depth.

In conclusion, we experimentally realize the longitudinal spin separation by modulating the PB phase. A beam with linear polarization changes its polarization into a space-variant one, and splits into two spin states with space-variant PB phase. The separation of the pair of spin states can be achieved and controlled by modulating the PB phase. Due to the spin-dependent divergence and convergence caused by setting the PB phase as a phase factor of a spherical wave, the focused Gaussian beam splits into two opposite spin states, and focuses at different distances, representing longitudinal spin separation. The performance of the longitude spin-dependent focal shift represents a novel manifestation of the spin-dependent separation, and adds an extra freedom for spin splitting. By combining this longitudinal spin separation with the transverse one, we experimentally achieve the controllable spin-dependent separation in three dimensional space. This work provides new insight on steering the spin photons, and is expected to be explored novel applications on optical trapping, and micromachining with higher degree of freedom.

## Additional Information

**How to cite this article**: Liu, S. *et al*. Longitudinal spin separation of light and its performance in three-dimensionally controllable spin-dependent focal shift. *Sci. Rep.* **6**, 20774; doi: 10.1038/srep20774 (2016).
